# Anatomical, Pathological, and Therapeutical Researches on the Yellow Fever of Gibraltar, of 1828

**Published:** 1839-12-07

**Authors:** 


### BIBLIOGRAPHICAL NOTICE.

*Anatomical, Pathological, and Therapeutical Researches on the Yellow Fever of Gibraltar, of* 1828. By P. Ch. A. Louis, D. M. P., &c. &c., *from Observations taken by himself and* M. Trousseau, *as Members of the French Commission at Gibraltar. Translated from the Manuscript, by* G. C. Shattuck, Jr., M. D. Boston: 1839.

This is the title of the last work of the celebrated Louis, and has first seen the light, in a country and in a language unfamiliar to its author. This event, singular in itself, is the result of circumstances highly honourable to those of our medical countrymen who have sought to perfect themselves in professional knowledge in Paris, and affords the strongest evidence of the paternal interest which M. Louis has taken in their pursuits, and of the personal esteem with which he has honoured some of the more zealous amongst them. Of the foreign medical students in Paris, the Americans have almost always been the assiduous followers of M. Louis; and there have usually been some one or more of them, who, by their superior industry, acquirements, and zeal, have not only attracted his attention, and secured his good offices, but also enjoyed the high privilege of his personal intimacy. Of this number was the translator of the present work. He had an opportunity, enjoyed by few others, of examining the original manuscript, and was so well convinced that its publication would confer an important benefit upon the scientific world, and especially upon those of our medical brethren in the South who are called upon every year to study and to treat cases of yellow fever, that he solicited M. Louis to make it public. Peculiar circumstances then operated, as they had done for nine years before, to prevent the satisfaction of this desire; but M. Louis at length consented to allow our countryman to translate and publish the work in the United States.

After the almost innumerable volumes written upon the history of yellow fever, in general, and concerning particular epidemics, it might, at first sight, appear superfluous to add a new treatise to the catalogue. But such an objection would have been equally plausible in regard to pulmonary affections and continued fevers, and would have continued so, had not a mode of observation, new in extent if not in principle, thrown a flood of light upon these two classes of diseases, and dissipated much of the obscurity in which they were enveloped. The same system of examination so successfully pursued by M. Louis in the study of phthisis and typhoid fever, has been applied to the epidemic of Gibraltar of 1828, and, we think, with hardly inferior results. As will be seen in the sequel, he has established, beyond the possibility of doubt, the anatomical lesion of the disease as it existed when observed by him, and has reduced to a certainty other points which the observations of all other writers had left in doubt. He has done this, not by any exertion of superior genius, not by any ingenious or analogical reasoning, but by patient study, unwearied industry, and, above all, by bringing to the investigation an unbiassed mind, a clear judgment, and a pure love of truth. A work conceived and executed in such a spirit cannot fail to have admirers wherever strict philosophical research is valued, and ought to be gratefully welcomed in a country where the dogmas of obsolete schools, and the mere opinions of illustrious men have lost the *prestige* which they still exercise in the old world.

In the year 1828 M. Louis was one of several physicians commissioned by the French government to ascertain the origin and the mode of propagation of the yellow fever then raging at Gibraltar. Besides fulfilling the immediate object of his appointment, he applied himself to a minute study of the disease, its pathological anatomy, its symptoms, and its treatment. He examined with great care the bodies of twenty-five persons who fell victims to the fever, besides noting the symptoms during life in these and in many other cases in which recovery took place. All the details concerning the former class of patients are given in the work under consideration, and several examples illustrative of the latter portion are subjoined.

He commences by narrating, in full, the history of four fatal cases, taken at random, in order to familiarize the reader with the general portraiture of the disease; he then gives an accurate account of the appearances presented in each of the organs after death, devoting a separate chapter to each particular organ, and concluding this part of the subject by an abstract of the anatomical lesions, and of the proportion of the twenty-five cases examined, in which each was found. We proceed to give some of the more important portions of this abstract:

"The mucous membrane of the trachea was red, and a little softened in one case.

We found black spots, and generally many of them, through the thickness of the lungs, in nine subjects. They were of different dimensions, and the tissue surrounding them increased in density in most of the cases. This was sometimes entirely deprived of air, in consequence of the effusion of a greater or less quantity of blood more or less combined with air. In six cases we found in the lungs tumours of a blackish red colour, of an irregular form, containing no air, not granulated, more or less firm, without evident organization.

The œsophagus was completely deprived of epidermis through its whole length in a third part of the cases, and partially so in a greater number.

The stomach was larger than natural in seven subjects. It contained a clear or dark red coloured liquid, a blackish or perfectly black fluid, in different quantities, in three quarters of the cases. Its mucous membrane was red through a greater or less extent in six cases; rose coloured or orange in eight cases; grayish, yellowish, or whitish in the others. It was thickened through a greater or less extent of surface in half the cases; softened and yellow to an extreme degree in the same number; at the same time thickened, softened and red in a third part of the cases; mamelonated in two thirds; ulcerated in two cases. It was natural in five cases.

The mucous membrane of the duodenum was red in a little more than half the cases, softened in the same number, and thickened in one case.

The small intestine contained a greater or less quantity of reddish, brownish, blackish, or perfectly black matter, in two-thirds of the cases. Its mucous membrane was slightly injected, or red in spaces, in a little less than half the cases. Its consistence was more or less diminished through its whole length, or through a part of its extent only, in rather a greater number of cases. It was partially thickened in one case, in no case was it ulcerated, and Peyer's glands were always natural.

The large intestine was of a greater size than usual in two cases. In fifteen cases it contained a matter of wine lees colour, or blackish, or brownish, or chocolate coloured, or entirely black. Its mucous membrane was of a pale or bright red colour in five cases, grayish, yellowish, or whitish in the others. Its consistence was more or less diminished in three-quarters of the subjects. Its thickness was increased in three cases, and twice we found it slightly ulcerated.

The liver was of a greater size than natural in two cases; a little firmer than usual in three cases, a little less firm in three others. Its cohesion was increased in six cases, diminished in seven. *Its colour was altered in every case*, being sometimes of the colour of fresh butter, sometimes of a straw colour, sometimes of the colour of coffee and milk, sometimes a yellowish gum colour, or, finally, sometimes an orange or pistachio colour. This discolouration was not the same through the whole extent of the liver, being more marked in the left than in the right lobe; it was also more uniform. In cases where the colour was uniform in the left lobe, there was in the right lobe a mixture of gum yellow, orange or red points, larger or smaller, or else we found in the right lobe a rose tint, which did not exist in the left lobe. With the discoloration of the liver, we found a more or less marked paleness, and a diminished quantity of blood, so that wherever this appearance of the liver was well marked, the sections of it were dry, and of an arid appearance in the left lobe. This appearance reminded us at first of the greasy transformation of the liver, a transformation always accompanied by a softening, more or less marked. In the cases now under consideration, the cohesion of the liver was not at all diminished, even where this organ was of a clear coffee and milk colour, or of a straw yellow, or of the colour of sole leather."

From these quotations it is evident that the essential anatomical character of the yellow fever of Gibraltar, in 1828, was the alteration of the colour of the liver, while the inflammation of the gastric mucous membrane must be considered as common to this disease and many others. In none of the lesions discovered after death, does M. Louis find the means of explaining either the symptoms observed during life, or the cause of death. None of the organs, the brain, the lungs, or the heart, the integrity of whose functions is usually considered necessary to life, were found disordered in many of the cases, and, even when altered, were only so in a very moderate degree. This absence of lesions sufficient to explain the cause of death, says M. Louis, must be admitted as "one of the characteristics of the yellow fever, at least of that which prevailed epidemically at Gibraltar in 1828, and if observation shows that there is in disease something beyond what we see, that its cause has sometimes a great deal to do with the death of those who are attacked by it, this double proposition is more evident here, where we must admit that the cause of the disease often kills by itself, or independently of any appreciable alterations of the organs, and even up to a certain point, derangement of the functions. We must remember that nearly the same thing happens in certain cases of poisoning."

The above sentence which expresses an idea frequently repeated throughout the work, fully exculpates M. Louis from the charge which has been made against him of attaching an undue importance to the connexion between the symptoms and termination of a disease, and the lesions which characterize it; in other words, of holding morbid anatomy to be rather the foundation of the history and treatment of diseases, than their exponent. It is only by long and discriminating study that one can become capable of deciding what share of influence an organic lesion has had in producing death; hence we should adopt a treatment founded upon such a study with becoming caution, for, as M. Louis observes, while "an intimate knowledge of the lesions observed in yellow fever is indispensable to the practitioner, he must also remember, that in this disease, as in others, there is something besides the lesions, and that he must profit by all that experience or chance may teach him of the best mode of treatment."

The author next proceeds to a study of the symptoms of yellow fever, as occurring in the epidemic in question, and devotes to the consideration of each one a separate chapter, besides describing the varieties of it met with in fatal, severe, and mild cases respectively. The limits prescribed to this notice forbid us from entering into a minute detail on this portion of the subject, we shall therefore content ourselves with borrowing the concise description of the symptoms given in the chapter on diagnosis. And, first, of the severe cases:

"In the most common cases of this kind, the patients experienced, at the commencement, headach; a pricking sensation in the eyes, which became almost immediately red and suffused; pains in the limbs, and more or less marked febrile symptoms. These symptoms continued. To them were joined pains in the epigastrium; nausea; spontaneous vomitings, some twelve or fifteen hours afterwards, rarely earlier, and sometimes even later. They were accompanied by an anxiety very variable in degree, so that some of the patients were constantly restless, and could not remain quiet in any posture, at the same time their intellectual faculties not being deranged. On the third or fourth days of the disease the eyes became yellow, the skin was of a similar colour, the injection of the conjunctiva diminished, and the stools more blackish. If the disease terminated in recovery, the stools resumed their natural colour, the vomitings became less frequent, or ceased entirely, and with them the pains and uncomfortable feelings. In fatal cases the vomitings continued, they became more or less blackish, or completely black, the dejections were of the same colour, the yellowness increased, the heat forsook the limbs, and from twenty to thirty-six hours after the supervention of this state of things, sometimes sooner, sometimes later, that is to say, four, or five, or six days after the commencement of the disease, the patient died."

The mild cases were remarkable not only for the benignity, but for the smaller number of the symptoms, and the extremely short duration of the disease.

"Most commonly at the commencement there were headach; chills, followed by a slight degree of heat; pains in the limbs; redness of the face and eyes. The epigastric pains were rare, and so too were the vomitings, which were never spontaneous, and which in no case were of a brownish colour. The heat and the thirst were moderate, and so slight was the diminution of the strength, that the patients did not either keep their bed at all, or were there for half a day only, thus, according to their expressions, going through with the disease on foot."

According to the observations of the French commission, the mortality during the epidemic, was in the proportion of one to six and a half. The proportion of deaths amongst the children was smaller than amongst the women, and these in their turn suffered less from the fever than the men. But this statement is not given as applicable to the mortality of the yellow fever in general, nor even to that of previous epidemics at Gibraltar.

In the chapter on diagnosis M. Louis exhibits a parallel between the symptoms of yellow and of typhoid fever, and has not much difficulty in establishing their distinctive characters. It was natural for him thus to compare the yellow fever with almost the only continued fever which he had before had an opportunity of observing. How much is it to be regretted that he had never been in a situation to study the diseases of our southern states, or of other regions where similar endemics occur, that he might have compared their features with the characters of yellow fever, and established whether or not their family likeness to that affection entitled them to be ranked amongst its varieties. Here is a field for those of our southern countrymen who aspire to a high rank in the medical world. They have a path marked out for them, in the admirable work before us; the plan of their studies accurately defined, and the encouragement of being able to produce a history of yellow fever, which shall for ever set at rest the numerous questions in regard to it, which now puzzle the inquirer after truth, and give rise to interminable discussions about the nature and distinctive marks of that disease. Careful and systematic observations, an accurate record of them, and a logical analysis of their facts, are all that is necessary to accomplish more brilliant discoveries, and to confer a more lasting benefit on the scientific world, than any hitherto attained.

M. Louis gives some very interesting statements in answer to the question, "Does a first attack of yellow fever preserve from a second?" It results from an examination of a vast mass of evidence upon this subject, that, "among nine thousand persons, who had once been attacked by yellow fever, and who had been exposed a second time to the influence of all the causes of that disease, twenty-five had it twice." He therefore concludes "that a first attack of yellow fever preserves from a second, as effectually, and at least in as great a degree, as a first attack of an eruptive disease, of the small-pox for instance, preserves from a second attack of the same disease."

The treatment made use of in the epidemic of Gibraltar was of two kinds, according as the patients came under the care of the English physicians of the post, or under that of the Spanish physicians of the city. The former class of practitioners had recourse, in the beginning of the epidemic, to very free depletion, regarding it as their principal therapeutic agent, but afterwards restricting its office to that of an auxiliary to purgatives, and large doses of calomel. In the commencement of an attack they would prescribe eight grains of jalap, and as much of scammony and calomel, and follow the administration of these agents by that of an ounce or an ounce and a half of castor-oil. In addition, purgative enamata were prescribed, and repeated on the following days, together with an ounce of castor-oil, or epsom or glauber salts by the mouth. "All, or nearly all, prescribed calomel, at the dose of from two to three grains every two hours, counting from the first or second day of the disease." "After the first week of the epidemic, the practice of the military medical men became the same; they all administered purgatives and calomel, regarding the latter as their sheet anchor. At this time blood-letting was used only to combat particular symptoms, such as pain at the epigastrium, headach; and against this last leeches were much used. Under the same circumstances blisters were applied to the epigastrium, to prevent the recurrence of vomitings, and at the same time Riviere's anti-emetic potion was prescribed, or else some preparation of opium."

"In the town of Gibraltar, little use was made of mercurial preparations, except in severe cases. Unprofessional persons thought they had found in olive oil an almost infallible remedy. A considerable dose of it was taken, and an enema to promote its operation, but no other remedies were employed. A Spanish physician, Dr. Ardevol, gave, in the commencement of the disease, a mild purgative, and if, in the first six hours of the disease, there was obtuse pain or epigastric oppression, an emeto-cathartic. He considered this treatment inefficient or even injurious, later in the disease. During the first twenty-four hours he prescribed emollient enemata every two or three hours, and advised abstinence from food, and the use of acidulated drinks. When there were strong symptoms of gastritis, he excited the action of the bowels by means of laxative enemata; and when the patient became feeble, he prescribed a draught in which the distilled water of orange flowers and lemon peel were the principal ingredients, or an infusion of quinquina. He had recourse to mercury in desperate cases only, prescribing frictions with two ounces and a half of mercurial ointment; and if on the third day, with a commencement of salivation, there was neither hiccough nor yellowness, the cure was certain. But, all things considered, the mortality was equal in the two classes of patients so differently treated."

Upon this startling, but unavoidable conclusion, M. Louis makes the following remark, which might serve as a motto to all who are not to be deterred from adopting a logical inference from well ascertained facts, because it may happen to war with previous opinions, or instinctive doubts. "Undoubtedly, these results will appear very singular, and for that reason there may be some who will refuse to admit them. BUT SCIENCE IS NOT AN AFFAIR OF SENTIMENT; WE SHOULD ASK ONLY WHAT IS TRUE, AND WHETHER CONCLUSIONS ARE RIGIDLY DEDUCED."

The author proceeds to inquire what indications are to be fulfilled in the treatment of yellow fever. He reminds the reader that in the majority of the cases there was gastritis, in all an affection of the liver of an unknown nature, and that the severest symptoms could be referred neither to these nor to any other lesions. "These facts show that we can only hope for a moderate success, even by the best adapted treatment, since we can direct our means against a part of the trouble only, that which is apparent. These facts are to be borne in mind for another reason; they do not warrant the rejection of an empirical treatment, however absurd or ridiculous it may appear, since it is against an unknown cause that therapeutic agents are to be employed." In the absence of any treatment, the utility of which had been demonstrated by experience, M. Louis recommends the following, as pointed out by a study of the symptoms and lesions.

He thinks that in the first twenty-four hours bleeding should be employed, the amount of blood withdrawn to be regulated by the severity of the febrile symptoms. The patient should take cool and slightly acid drinks in as large a quantity as the stomach will bear without nausea. The intestinal canal should be evacuated by mild enemata, repeated several times in the twenty-four hours, and emollient fomentations should be applied over the abdomen. He discusses the propriety of applying leeches to the epigastrium to relieve the pain there, and to control the vomiting, and questions the superiority of this mode over general depletion. He doubts the probability of arresting the intestinal hæmorrhage by venesection, and suggests the employment of astringents before the appearance of the black stools. In regard to the remedy proper to alter the morbid condition of the liver, he says its discovery "must be left to time and chance, and to the acuteness of the observer, for experience has sufficiently proved that no dependence is to be placed on mercurial preparations of any sort."

In concluding this brief account of a work of which no analysis can give a just idea, we cannot refrain from expressing a hope that its general perusal may excite American physicians to avail themselves of the ample materials presented every year for a thorough investigation of the nature and characters of yellow fever. In no other country where the disease occurs epidemically, is medical science better cultivated than in this; it is, therefore, our privilege, as well as our duty, to examine for ourselves, and to rest contented no longer with the theories of the past, no matter how ingenious or plausible, and still less to be satisfied with the crude and uncertain opinions derived from what is so fondly and so falsely called "experience."

